# Associations of In Utero Exposure to Racial Violence and Reproductive Development: The Bogalusa Heart Study

**DOI:** 10.1002/ajhb.70163

**Published:** 2025-10-25

**Authors:** Maria P. Santos, Maya David, Lydia Bazzano, Katrina Sims, Emily W. Harville

**Affiliations:** ^1^ Department of Epidemiology Tulane University School of Public Health and Tropical Medicine New Orleans Louisiana USA; ^2^ Tulane University School of Medicine New Orleans Louisiana USA; ^3^ Department of History Hofstra University Hempstead New York USA

**Keywords:** civil rights, menarche, prenatal stress, race, violence

## Abstract

**Objective:**

This study seeks to assess the association between in utero exposure to racial violence during the Civil Rights movement and pubertal development and fertility outcomes within the Bogalusa Heart Study population.

**Methods:**

Utilizing a prospective cohort design, Bogalusa Heart Study participants born between 1960 and 1970 were categorized based on their gestational age during peak racial violence events in Bogalusa. Exposure was defined as being in utero during the first trimester during February–July 1965. Pubertal development was assessed using age at menarche for girls and Tanner staging at age 13 for boys (*n* = 1945) and girls (*n* = 1970). Fertility outcomes, including fertility issues and miscarriage, were obtained by self‐report from the Bogalusa Babies study (2012–2016).

**Results:**

In utero exposure to racial violence was associated with earlier age at menarche in girls (−0.43 years, *p* < 0.001) and delayed pubertal development in boys (−0.54 Tanner stage at age 13; *p* = 0.02). An imprecise estimated increased odds of miscarriage (OR = 2.02, 95% CI: 0.92 to 4.47) and fertility issues (OR = 2.66, 95% CI: 0.62 to 11.32) were observed. Analysis by race did not show a significant interaction.

**Conclusion:**

In utero exposure to racial violence during the first trimester of pregnancy was associated with an earlier age at menarche in girls and slower pubertal development in boys. The findings underscore the importance of considering maternal stressors, specifically racial violence, in understanding variations in reproductive development.

## 1 Introduction

Exposure to violence, especially when infused with racial elements like police‐related fatalities, has been linked to detrimental health outcomes, including reproductive impacts such as reduced birth rates, heightened occurrence of low birthweight, and increased sexually transmitted infections (Ibragimov et al. 2020; DeVylder et al. 2018; Chandran et al. 2021; Jahn et al. 2021). Such encounters also exert profound psychological stress, particularly evident within Black families (DeVylder et al. 2018; Wise et al. 2009). Exposure to violence during the prenatal and early childhood phases has been associated with modifications in the maturation process of both boys and girls, as well as subsequent effects on adult fertility (Wise et al. 2009; Suglia et al. 2020; Magnus et al. 2018). These stressors might exert lasting effects on the endocrine system or induce changes in DNA methylation and other epigenetic factors (Heim et al. 2001; Yam et al. 2015; Tyrka et al. 2012; Rubens et al. 2023). The first trimester is recognized as a particularly sensitive period in fetal development, especially in the programming of neuroendocrine and reproductive systems. Maternal stress early in pregnancy may influence the fetal hypothalamic‐pituitary‐adrenal (HPA) axis and subsequent developmental trajectories. Prior studies have demonstrated that elevated maternal cortisol in early gestation is linked to adverse developmental outcomes, providing a theoretical basis for focusing on first‐trimester exposure (Wise et al. 2009; Suglia et al. 2020; Magnus et al. 2018; Davis and Sandman 2010).

In the 1960s, residents of Bogalusa, Louisiana experienced several exposures to racial violence as the area emerged as a prominent center of both the Civil Rights Movement and reactionary racial violence (Table S1). This period witnessed the significant presence of the Deacons for Defense, a key part of the Black armed resistance movement. Multiple incidents of cross burnings occurred in 1964. In 1965, the intervention of the Louisiana governor was required to prevent Klan‐related violence in the region. Tragically, during 1965–6, a Black sheriff's deputy and a participant in civil rights marches were killed; however, the perpetrators were never convicted (Fairclough 2008).

This study seeks to investigate the connection between in utero exposure to violence and social unrest surrounding the Civil Rights movement and reproductive development. We hypothesized sex‐specific effects based on literature suggesting differential impacts of prenatal stress on the hypothalamic–pituitary‐gonadal axis. Girls may experience earlier pubertal onset in response to psychosocial stress via alterations in HPA axis functioning and metabolic programming, while boys may exhibit delayed maturation due to stress‐induced disruptions in the hypothalamic–pituitary‐gonadal (HPG) axis and testosterone production (Bale and Epperson 2015; Koss and Gunnar 2018). These pathways reflect sexually dimorphic developmental plasticity and align with the Developmental Origins of Health and Disease (DOHaD) framework. The aims of this study are to investigate the correlation between exposure to race‐related civil rights disturbances during prenatal stages and (1) the timing of pubertal development and (2) subsequent offspring adult fertility.

## 2 Methods

### 2.1 Study Population

The Bogalusa Heart Study (BHS) is a longitudinal study started by Dr. Gerald S. Berenson that focuses on studying the early development and progression of cardiovascular disease (CVD). The BHS began in 1973 and has followed individuals from childhood to adulthood in the semi‐rural community of Bogalusa. The initial participants, aged 3–18, approximately 65% White and 35% Black, were selected from local schools, and additional participants have been added to the study over time. Data collection has taken place at intervals of approximately every 2 years for children and every 5 years for adults. The findings from these periodic assessments have been combined to form the overall study population of the BHS.

### 2.2 Exposure

The dataset was limited to those born between 1960 and 1970 (Table 1). Each participant was categorized based on their birthdate and gestational age at birth (taken from birth certificates) at the time of peak events of racial violence in Bogalusa. Trimester‐specific exposure was defined using obstetric‐estimate gestational age: first ≤ 12 weeks, second 13–27 weeks, third ≥ 28 weeks. Each birth belongs to one, and only one, trimester category. If participants were in utero during their first trimester (up to 12 weeks) between February and July 1965, they were considered to be exposed. We chose February–July 1965 as the peak period of racial violence events during the civil rights era because, during this period, there were clashes between civil rights activists (CORE) and the KKK until a federal judge required enforcement of federal civil rights laws by police. We then defined less violent times before and after this time. A timeline of the events that took place in the area during the civil rights era can be found in the Table S1.

**TABLE 1:** Participant sample characteristics by exposure group.

	In utero before or after peak periods^a^	In utero (first trimester) Feb–July 1965	*p*	*N* missing
*N*	2078	71		
Race (%)				11
White	1317 (64)	42 (60)	0.43	
Black	750 (36)	29 (40)		
Age at Menarche, mean (SD)	12.11 (1.5)	11.55 (1.8)	0.01	147
*n*	1957	45		
Tanner stage at age 13 (girls)	3.5 (1.0)	4.2 (1.2)	0.01	179
*n*	1931	39		
Tanner stage at age 13 (boys)	3.8 (1.2)	3.1 (0.9)	0.02	204
*n*	1903	42		
Any PCOS (%)				25
Yes	163 (8)	1 (2)	0.03	
No	1890 (92)	70 (98)		
Any fertility issues (%)				0
Yes	379 (18)	6 (9)	0.04	
No	1699 (82)	65 (91)		
Any miscarriage (%)				0
Yes	662 (32)	31 (44)	0.03	
No	1416 (68)	40 (56)		
Not pregnant (> 12 months)				975
Yes	501 (44)	19 (52)	0.09	
No	572 (51)	17 (48)		
Not sure	66 (5)	0		
Childhood BMI, mean (SD)	16.8 (2.2)	16.1 (1.8)	0.02	0
*n*	2078	71		
Adult BMI, mean (SD)	31.9 (0.3)	29.4 (1.3)	0.15	0
*n*	2078	71		
Smoke				1174
Yes	297 (31)	16 (68)	0.46	
No	635 (69)	27 (37)		
Drink				1527
Yes	190 (31)	9 (43)	0.24	
No	411 (69)	12 (57)		
Home ownership (family)				867
Yes	920 (75)	27 (57)	0.03	
No	227 (18)	16 (34)		
Don't know	88 (7)	4 (9)		
Education				861
< High school	165 (13)	7 (15)	0.02	
High school	461 (37)	23 (49)		
Some college	320 (26)	14 (30)		
Bachelor's or higher	295 (23)	3 (6)		
Adverse Childhood Experience score, mean (SD)	2.1 (2.2)	3.3 (2.4)	0.71	942
*n*	1152	55		
Own birth weight, mean (SD)	3134 (619)	2998 (713)	0.19	1037
*n*	1062	50		

^a^ These dates include participants born between January 1, 1960, and December 31, 1970.

### 2.3 Outcomes

Pubertal development was defined as the age of menarche for girls and Tanner stage at age 13 for boys and girls. Tanner staging assessments were performed by trained pediatric clinicians during in‐person Bogalusa Heart Study physical exams. Tanner staging is an objective classification system used for the development of secondary sex characteristics in children during puberty. Tanner staging includes two scales for each sex: a pubic hair scale (both males and females), a breast development scale (for females), and an external genitalia scale (for males); specific guidelines on how each stage within these scales is available in the Table S2. We used the average of the two scales to define the Tanner stage at age 13 for participants.

Fertility and miscarriage were collected from female participants with a mean age of 50.3 years (SD = 6.4; range: 36–58 years), indicating likely completed fertility, as part of the Bogalusa Babies study, conducted in 2012–2016. Female participants (*n* = 1803) were interviewed about their reproductive history. More information on the study design of the Bogalusa Babies study is detailed elsewhere (Delemarre‐van de Waal et al. 2002). Fertility was defined as having taken any fertility drugs or received any medical procedures to help them get pregnant; ever having tried to become pregnant and not been able to for at least 12 months; or having ever been to a doctor to get help in getting pregnant. Self‐report data on any miscarriage (yes/no) was also used as an outcome measure of fertility. While self‐report has limitations, it provides a reasonable estimate of fertility difficulties such as use of fertility treatment and long time to pregnancy (Hvidtjorn et al. 2009; Cooney et al. 2009; Jukic et al. 2016).

### 2.4 Covariates

Covariates included possible confounders and effect modifiers in the relationship between in utero exposure to racial violence and reproductive development. Race in this context is not considered a biological determinant but a social construct that captures differential exposure to structural and interpersonal racism, which can influence reproductive health through psychosocial stress and unequal access to resources. Initially, data were extracted for risk factors such as assigned race (White or Black), self‐reported polycystic ovarian syndrome (PCOS; yes or no), childhood BMI (continuous), adult BMI (continuous), smoking, drinking, home ownership of family (yes, no, unknown), education (< high school, high school graduate, some college, Bachelor's degree or higher), adverse childhood experience (ACE) score (continuous), own birthweight (continuous), and uncontrolled diabetes (yes, no). Variables presented a missingness pattern of missing completely at random (MCAR). Directed Acyclic Graphs were created to identify potential confounders for the puberty outcomes (Figure S1) and for fertility outcomes (Figure S2) and guide covariate inclusion in our models. While no adjustment was deemed necessary to estimate the overall impact (total effect) of exposure to peak times of racial violence during the first trimester in utero on pubertal development and fertility, we did include them in our adjusted model to specifically gauge whether this exposure was associated with the outcomes independent of potential intermediary variables.

### 2.5 Statistical Analysis

For continuous outcomes, age at menarche and Tanner staging, we computed Least Squares (LS) means to adjust for covariates and assess group differences. For binary outcomes, such as miscarriage and fertility issues, we used logistic regression to calculate odds ratios (ORs), considering potential confounders. We addressed missing data using a complete case analysis approach, as the variables presented a missingness pattern of MCAR. There was no missing data for fertility problems, miscarriage, or adult BMI or childhood BMI by design; PCOS was missing for 1%, race less than 1%, age at menarche 7%, Tanner staging for girls had 8% missing, and Tanner staging for boys had 9% missing.

We tested interactions and performed subgroup analysis by race as we hypothesized race could modify the relationship between exposure to racial violence and reproductive development.

We conducted additional sensitivity analyses comparing exposure during the second and third trimesters and for any exposure during pregnancy (February–July 1965). We also compared models using alternative control periods, including pre‐1965 and post‐1965 births, to assess the robustness of findings. Furthermore, we adjusted for the season of birth (categorized as winter, spring, summer, fall) to account for potential confounding by seasonal effects on developmental timing.

### 2.6 Ethics Approval

The Bogalusa Heart Study and Bogalusa Babies studies were approved by the Tulane University Institutional Review Board.

## 3 Results

### 3.1 Age at Menarche

Mean age at menarche in female individuals exposed to racial violence during the first trimester in utero was 11.69 years (95% CI: 11.31 to 12.08), whereas those not exposed had a mean age of 12.12 years (95% CI: 12.04 to 12.20; *p* < 0.001). Similar trends were observed in both Black and White girls in subgroup analysis (Table 2). No significant interaction with race was found.

**TABLE 2:** LS means (95% CI) for age at menarche and Tanner staging.

Group	Outcome	In utero during peak times	In utero before/after peak	*p*	*N* (exposed/unexposed)
Full sample	Age at menarche^a^	11.69 (11.31, 12.08)	12.12 (12.04, 12.20)	< 0.001	45/1886
	Tanner staging (girls)^a^	3.61 (3.52, 4.24)	3.56 (3.41, 4.08)	0.34	42/1857
	Tanner staging (boys)^b^	2.54 (1.93, 2.92)	3.08 (2.92, 3.47)	0.02	39/1829
White only	Age at menarche^a^	11.80 (11.34, 12.31)	12.21 (12.11, 12.31)	< 0.001	28/1272
	Tanner staging (girls)^a^	3.67 (2.53, 3.82)	3.07 (2.54, 3.60)	0.92	26/1258
	Tanner staging (boys)^b^	2.89 (2.27, 3.39)	3.01 (2.82, 3.48)	0.07	25/1235
Black only	Age at menarche^a^	11.53 (10.93, 12.18)	12.01 (11.87, 12.14)	< 0.001	17/614
	Tanner staging (girls)^a^	3.89 (3.69, 4.10)	3.68 (3.43, 4.02)	0.82	16/599
	Tanner staging (boys)^b^	2.75 (2.10, 3.29)	3.09 (3.19, 3.57)	0.06	14/594

^a^ Adjusting for childhood BMI and any PCOS.

^b^ Adjusting for childhood BMI.

### 3.2 Tanner Staging

For Tanner staging in girls at age 13, those exposed to racial violence during the first trimester in utero had a mean Tanner stage at age 13 of 3.61 (95% CI: 3.52 to 4.24), while those not exposed had a mean Tanner stage at age 13 of 3.56 (95% CI: 3.41 to 4.08; *p* = 0.34). In boys, the exposed group had a mean Tanner stage at age 13 of 2.54 (95% CI: 1.93 to 2.92), while the non‐exposed group had a mean Tanner stage at age 13 of 3.08 (95% CI: 2.92 to 3.47; *p* = 0.02). Similar trends were observed in both Black and White children in subgroup analysis (Table 2); no significant interaction with race was found.

### 3.3 Fertility Issues and Miscarriage

The odds ratio (OR) for the association between exposure to racial violence during the first trimester in utero and the likelihood of fertility issues was 2.66 (95% CI: 0.62 to 11.32) (Table 3). No interaction with race was found. For the occurrence of any miscarriage among individuals exposed to racial violence during the first trimester in utero, the odds ratio was 2.02 (95% CI: 0.92 to 4.47). No interaction with race was found.

**TABLE 3:** Odds ratios (95% CI) for self‐reported fertility issues and any miscarriage.

	OR	95% CI	*p*
Full sample			
Fertility issues	2.66	(0.62, 11.32)	0.18
Any miscarriage	2.02	(0.92, 4.47)	0.08
White participants only			
Fertility issues	1.88	(0.42, 8.32)	0.27
Any miscarriage	1.76	(0.64, 4.81)	0.26
Black participants only			
Fertility issues	1.02	(0.11, 14.09)	0.98
Any miscarriage	2.65	(0.72, 9.78)	0.08

*Note:* Adjusting for BMI and any PCOS.

### 3.4 Sensitivity Analysis

To evaluate the robustness of our findings, we conducted several sensitivity analyses. First, we assessed the impact of exposure during the second and third trimesters, as well as any in utero exposure during the peak violence period. The patterns of association were consistent with our main findings, though effect estimates were somewhat attenuated in later trimesters (Table S3). We also examined whether results varied depending on the reference group by comparing those exposed in the first trimester to births before 1965 and after 1965 separately. These comparisons yielded similar trends, suggesting our findings are not dependent on the chosen control window (Table S4). Finally, to account for possible confounding by seasonality, we adjusted for season of birth. Associations between in utero exposure and earlier age at menarche and delayed pubertal development in boys persisted after this adjustment (Table S5).

## 4 Discussion

Our study indicates that exposure to racial violence during the first trimester in utero was associated with significant differences in age at menarche and Tanner staging in 13‐year‐old boys. In terms of fertility and fecundability, there was no statistically significant association between exposure to racial violence during the first trimester in utero and the risk of fertility issues or any miscarriage. Although our analysis focused on the first trimester due to its known biological sensitivity, our sensitivity analyses suggest that exposure across pregnancy may also influence reproductive development. The persistence of associations even when using broader exposure windows enhances confidence in the observed relationships.

Similar studies have shown that maternal stressors can be prenatal determinants of reproductive development. A study from Quebec (Dick et al. 2003) analyzed the association between maternal stress and age at menarche. They found that childhood BMI mediated the association between maternal stress and earlier age at menarche. A more recent study (Duchesne et al. 2017) discovered a non‐linear U‐shaped association between maternal stressful life events and earlier age of menarche in their offspring. European studies have also found associations between being exposed in utero to environmental stressors such as the Great Depression of the 1930s, the interval of World War II, and the interval of communist rule following World War II through the 1950s, and lower ages at menarche (Bräuner et al. 2021; Sekajová et al. 2022; Liczbińska et al. 2018). These findings stress the connection between psychosocial factors and health outcomes, and the need for emphasizing women's mental health and support systems. Similarly, the healthcare system needs to find better ways to support pregnant women of lower socioeconomic status, who have fewer resources for mental health. However, the larger picture is the need to reduce exposure to violence and racism for the health of all.

Potential mechanisms underlying these associations could involve the impact of maternal stress on fetal development. Stress experienced by pregnant women, particularly from exposure to traumatic events like racial violence, can trigger hormonal changes and affect placental function, leading to alterations in fetal development (Liczbińska et al. 2023). These alterations may manifest as disruptions in hormonal signaling pathways involved in pubertal development and reproductive function later in life. Furthermore, stress‐induced changes in gene expression and epigenetic modifications (Yam et al. 2015; Tyrka et al. 2012; Rubens et al. 2023) could also play a role in shaping long‐term health outcomes in offspring. Our findings of divergent pubertal timing by sex align with theories of sexually dimorphic developmental sensitivity to early‐life stress. Girls may accelerate pubertal development in response to prenatal stress, a pattern possibly shaped by adaptive calibration of reproductive timing in response to environmental cues (Murphy et al. 2006). In contrast, boys may experience developmental delays due to the vulnerability of androgenic pathways to stress‐related HPA axis disruptions. This pattern is consistent with recent findings observing sex‐specific associations between maternal stress in pregnancy and pubertal timing in a large cohort study (Sutherland and Brunwasser 2018). However, other studies found no sex differences, suggesting that these effects may depend on the type, timing, or intensity of stress exposure (Colich et al. 2020). More research is needed to determine whether these divergent findings reflect true sex differences or methodological variations.

Although a growing body of research has linked early life adversity to alterations in pubertal timing (Kubo et al. 2025; Howland et al. 2025; Ding et al. 2024), few studies have explored the specific impact of racial violence‐related stress during pregnancy. While previous research has examined the biological consequences of racial violence, including racially motivated attacks and systemic oppression, on outcomes such as telomere length, low birth weight, and stress biomarkers (Gaml‐Sørensen et al. 2024; Chae et al. 2014), few studies have explored how such stressors may influence the timing of pubertal development. Our study expands this literature by focusing on prenatal exposure to racial violence and its association with offspring pubertal timing, a developmental outcome with lasting implications for health. Our study is the first to explore the associations between in utero exposure to racial violence and pubertal development. Additionally, we were able to use prospectively collected pubertal timing data, minimizing the potential for misclassification of our puberty outcomes.

Limitations of our study include that the fertility and miscarriage outcomes were self‐reported, which could lead to misclassification; however, multiple validation studies of self‐report fertility outcomes find reasonable to excellent agreement with medical records and prospective data, and they have consistently found higher self‐report data to have higher specificities than sensitivities, meaning that if non‐differential classification were to be present, it would most likely underestimate the odds ratio calculated, giving more conservative estimates (Hvidtjorn et al. 2009; Dominguez 2011; Kristensen and Irgens 2000). Validation studies show self‐report of miscarriage is mixed; however, there is no other reasonable source for information about early miscarriages in a community cohort. Misclassification of gestational age from birth certificates is also a study limitation as gestational age was derived from birth‐certificate obstetric estimates without corroborating early ultrasound; however, the use of birth certificates to estimate gestational age has been validated to have high sensitivity and specificity (Cooney et al. 2009). Racial violence likely had community‐wide effects, potentially affecting white families via secondary stress or social tension. Although mechanisms may differ, this shared environmental exposure may explain the absence of significant effect modification by race. Additionally, the absence of observed differences between racial groups may reflect limited statistical power rather than a true lack of differential effect, particularly given the small number of Black children exposed in utero. Future studies with larger and more racially diverse cohorts are needed to clarify potential race‐specific effects and to assess how structural racism interacts with prenatal stress pathways.

## 5 Conclusion

In utero exposure to racial violence during pregnancy was associated with an earlier age at menarche in girls and slower pubertal development in boys.

## Conflicts of Interest

The authors declare no conflicts of interest.

## Supporting information


**Table S1:** Timeline of events in Bogalusa.
**Table S2:** Tanner staging clinical guidelines.
**Table S3:** LS means (95% CI) for Age at menarche and Tanner staging by trimester of exposure
**Table S4:** Control period sensitivity analysis
**Table S5:** Sensitivity analysis adjusting for season of birth
**Figure S1:** Directed acyclic graph for puberty outcomes.
**Figure S2:** Directed acyclic graph for fertility outcomes.

## Data Availability

The data that support the findings of this study are available on request from the corresponding author. The data are not publicly available due to privacy or ethical restrictions.
