# Supplementary material for: Associations of In Utero Exposure to Racial Violence and Reproductive Development: The Bogalusa Heart Study
Source: Am J Hum Biol. 2025 Oct 25;37(10):e70163. doi: 10.1002/ajhb.70163 (PMC12553303; doi:10.1002/ajhb.70163)
Supplement: Supplementary file 1 — Table S1: Timeline of events in Bogalusa. Table S2: Tanner staging clinical guidelines. Table S3: LS means (95% CI) for Age at menarche and Tanner staging by trimester of exposure Table S4: Control period sensitivity analysis Table S5: Sensitivity analysis adjusting for season of birth Figure S1: Directed acyclic graph for puberty outcomes. Figure S2: Directed acyclic graph for fertility outcomes. [file AJHB-37-e70163-s001.docx]

**Appendix**

**Supplementary Table 1:** Timeline of Events in Bogalusa
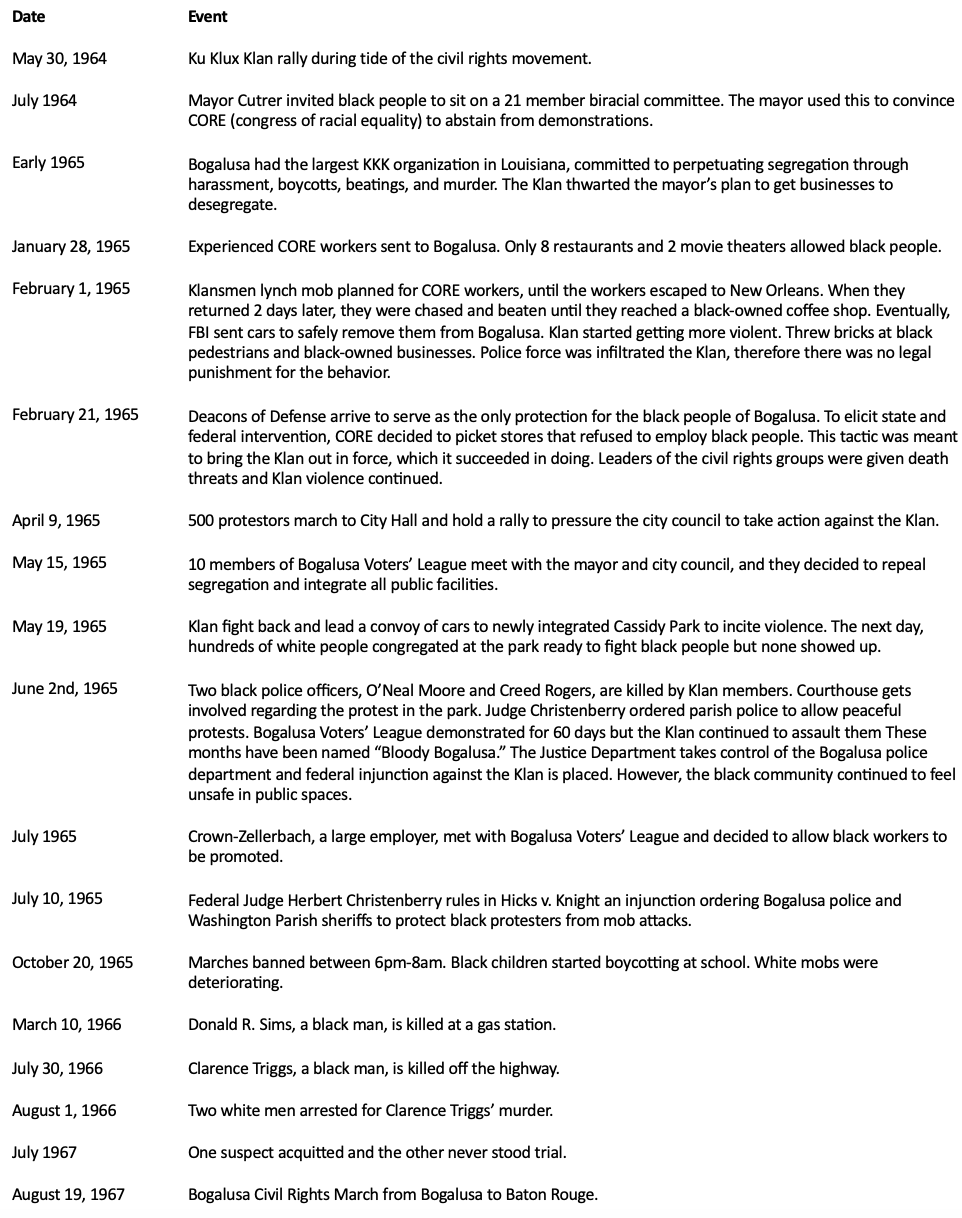


Adapted from *Race & Democracy: the civil rights struggle in Louisiana* [19]

**Supplementary Table 2:** Tanner Staging Clinical Guidelines

| Tanner Stage | Pubic Hair Scale | Female Breast Development Scale | Male External Genitalia Scale |
| --- | --- | --- | --- |
| Stage 1 | No Hair | No glandular breast tissue | Testicular volume < 4 ml or long axis < 2.5 cm |
| Stage 2 | Downy Hair | Breast bud under the areola | 4 ml-8 ml (or 2.5 to 3.3 cm long) |
| Stage 3 | Scant Terminal Hair | Breast tissue outside areola; no areolar development | 9 ml-12 ml (or 3.4 to 4.0 cm long) |
| Stage 4 | Terminal hair that fills the entire triangle overlying the pubic region | Breast tissue outside areola; no areolar development | 15-20 ml (or 4.1 to 4.5 cm long) |
| Stage 5 | Terminal hair that extends beyond the inguinal crease onto the thigh | Areolar mound recedes into single breast contour with areolar hyperpigmentation, papillae development, and nipple protrusion | > 20 ml (or > 4.5 cm long) |

**Supplementary Table S3:** LS Means (95% CI) for Age at Menarche and Tanner Staging by Trimester of Exposure

| **Exposure Group** | **Outcome** | **Exposed Mean (95% CI)** | **Unexposed Mean (95% CI)** | **P-value** |
| --- | --- | --- | --- | --- |
| 2nd Trimester Exposure | Age at Menarche* | 11.77 (11.30, 12.24) | 12.12 (12.04, 12.20) | 0.06 |
|  | Tanner Staging in girls* | 3.62 (3.50, 4.18) | 3.56 (3.41, 4.08) | 0.42 |
|  | Tanner Staging in boys^ | 2.66 (2.00, 3.02) | 3.08 (2.92, 3.47) | 0.11 |
| 3rd Trimester Exposure | Age at Menarche* | 11.91 (11.45, 12.33) | 12.12 (12.04, 12.20) | 0.37 |
|  | Tanner Staging in girls* | 3.70 (3.59, 4.25) | 3.56 (3.41, 4.08) | 0.30 |
|  | Tanner Staging in boys^ | 2.80 (2.10, 3.22) | 3.08 (2.92, 3.47) | 0.35 |
| Any Exposure During Pregnancy | Age at Menarche* | 11.73 (11.36, 12.10) | 12.12 (12.04, 12.20) | <0.01 |
|  | Tanner Staging in girls* | 3.66 (3.53, 4.21) | 3.56 (3.41, 4.08) | 0.25 |
|  | Tanner Staging in boys^ | 2.61 (2.02, 2.98) | 3.08 (2.92, 3.47) | 0.04 |

*Adjusting for childhood BMI and any PCOS
^Adjusting for childhood BMI

**Supplementary Table S4:** Control Period Sensitivity Analysis

| Referent Group | Outcome | Estimate | 95% CI | P-value |
| --- | --- | --- | --- | --- |
| Pre-1965 only | Age at Menarche | –0.42 years | (–0.69, –0.15) | <0.01 |
| Post-1965 only | Age at Menarche | –0.39 years | (–0.65, –0.12) | 0.01 |

**Supplementary Table S5:** Sensitivity Analysis Adjusting for Season of Birth

| **Outcome** | **Season-Adjusted Mean (Exposed)** | **Season-Adjusted Mean (Unexposed)** | **P-value** |
| --- | --- | --- | --- |
| Age at Menarche* | 11.66 (11.29, 12.03) | 12.10 (12.02, 12.18) | <0.001 |
| Tanner Staging (girls)* | 3.58 (3.49, 4.19) | 3.55 (3.40, 4.07) | 0.37 |
| Tanner Staging (boys)^ | 2.50 (1.91, 2.89) | 3.06 (2.91, 3.46) | 0.02 |

*Adjusting for season of birth, childhood BMI, and PCOS
^Adjusting for season of birth and childhood BMI


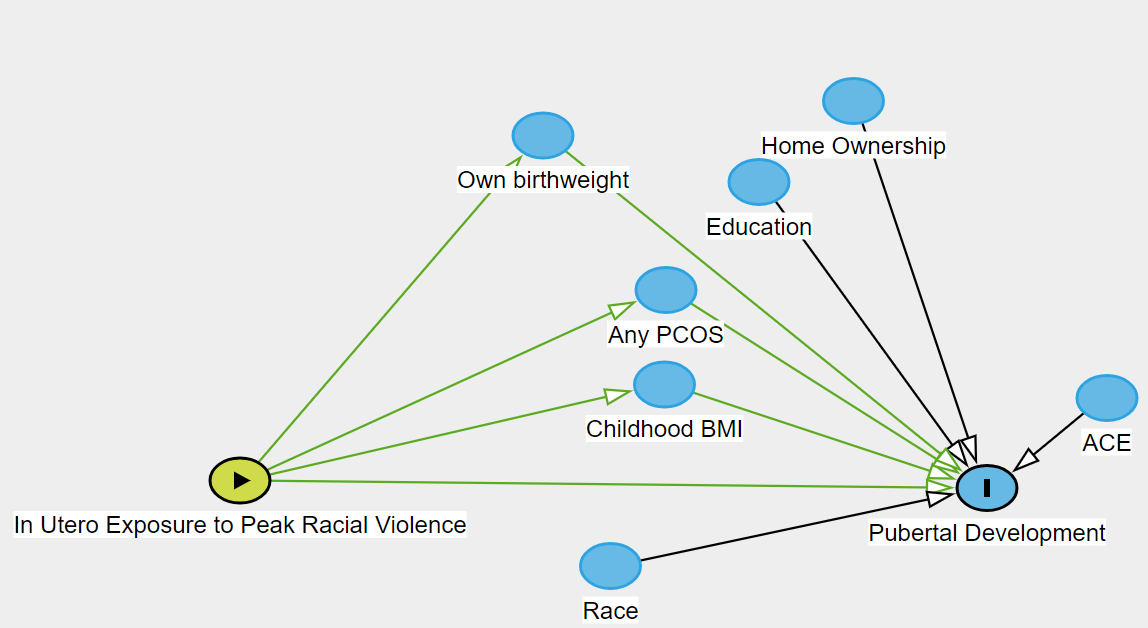


**Supplementary Figure 1:** Directed Acyclic Graph for Puberty Outcomes


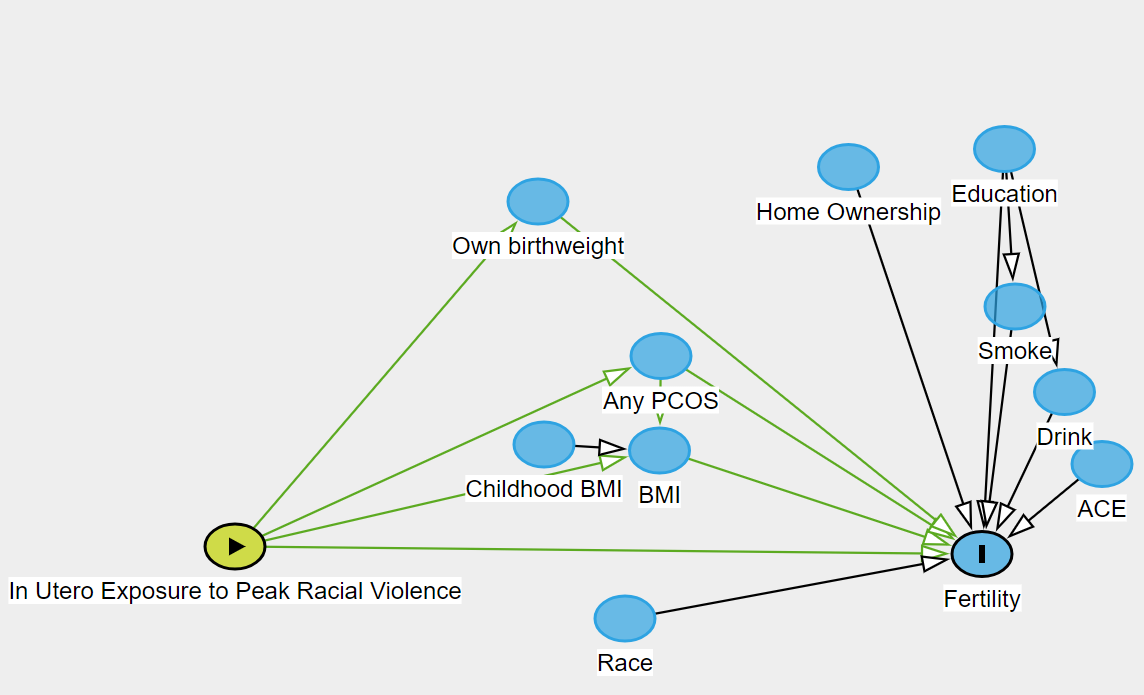


**Supplementary Figure 2:** Directed Acyclic Graph for Fertility Outcomes
